# Complete Genome Sequence of SMBL-WEM22, a Halotolerant Strain of Kosakonia cowanii Isolated from Hong Kong Seawater

**DOI:** 10.1128/MRA.00891-21

**Published:** 2021-10-14

**Authors:** W. E. Moore, G. K. K. Lai, S. D. J. Griffin, F. C. C. Leung

**Affiliations:** a Shuyuan Molecular Biology Laboratory, The Independent Schools Foundation Academy, Hong Kong SAR, China; University of Delaware

## Abstract

Kosakonia cowanii is a Gram-negative, motile, facultative anaerobic enterobacterium that is found in soil, water, and sewage. K. cowanii SMBL-WEM22 is a halotolerant strain that was isolated from seawater in Hong Kong. The complete genome of SMBL-WEM22 (5,037,617 bp, with a GC content of 55.02%) was determined by hybrid assembly of short- and long-read DNA sequences.

## ANNOUNCEMENT

Kosakonia cowanii is a Gram-negative, motile, facultative anaerobic enterobacterium that is found widely in soil, water, and sewage. It has been shown to protect against parasitic infection when present in the gut of mosquitoes (1–3). SMBL-WEM22 was isolated from a beach water sample from Repulse Bay in Hong Kong (22.2375884N, 114.1947159E) using 3M Petrifilm plates selective for Escherichia coli and coliforms.

Isolates were grown aerobically on Luria agar at 37°C and passaged eight times. A single colony, WEM22, was selected for genomic DNA extraction using an Invitrogen PureLink Genomic DNA Mini Kit. Paired-end short-read sequencing libraries were prepared using the Nextera XT DNA Library Preparation Kit and sequenced via the Illumina MiSeq platform with v3 chemistry (2 × 300 bp). Adapter sequences were removed using Trimmomatic v0.32 (4), and reads were quality filtered and trimmed with the following settings: LEADING:5, SLIDINGWINDOW:4:15, CROP:245, MINLEN:40. The resulting data set contained 830,287 read pairs (412.1 million bases), with an average length of 245 bp. Libraries for long-read sequencing were prepared using a genomic DNA Rapid Sequencing Kit and were sequenced using a SpotON Flow Cell (R9 version) and MinION sequencer, with data acquisition using MinKNOW v3.1.8 software and base calling with Guppy v2.1.3 (all from Oxford Nanopore Technologies). The final long-read data set, trimmed with Porechop v0.2.4 (5), totaled 880,157 reads (2 Gb), with a median read length of 1,217 bp and an *N*_50_ value of 4,536 bases.

Full genome assembly, by combining long-read and short-read DNA sequences using Unicycler v0.4.8 (6), yielded two replicons, i.e., a circular chromosome of 5,037,617 bp (GC content of 55.02%) and a plasmid of 157,340 bp (GC content of 51.52%), which were submitted to the NCBI Prokaryotic Genome Annotation Pipeline (PGAP) for annotation.

BLASTN v2.11.0 (7) analysis of the SMBL-WEM22 chromosome found 85 to 86% similarity to Kosakonia cowanii strains FBS 223, Esp_Z, and 888-76 (GenBank accession numbers CP035129.1, CP022690.1, and CP019445.1, respectively), and phylogenetic analysis based on 500 genes using RAxML v8.2.11 in PATRIC (8, 9) showed these to be the closest strains, with calculated average nucleotide identities of 93.06%, 93.04%, and 93.02%, respectively (10, 11). The SMBL-WEM22 chromosome incorporates 99% of the K. cowanii plasmid p888-76-2 (GenBank accession number CP019447.1), which is similarly integrated into the chromosomes of FBS 223 and Esp_Z.

**FIG 1:**
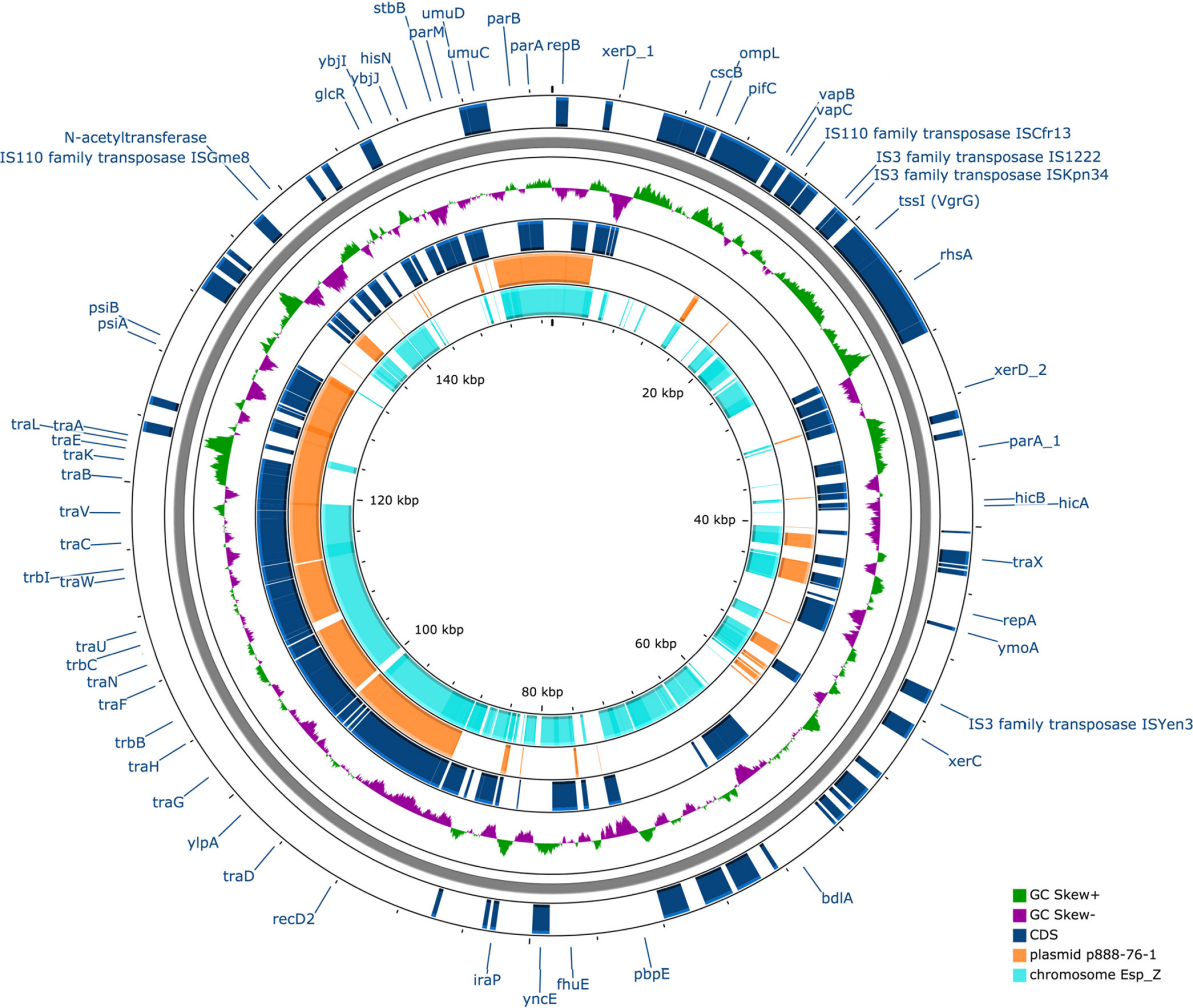
Plasmid pSMBL-WEM22 (157,340 bp) shows 37% and 51% alignment with plasmid 888-76-1 (orange) and the Esp_Z chromosome (light blue), respectively. Map generated by CGView (21).

BLASTN analysis of plasmid pSMBL-WEM22 showed 37% and 51% alignment with plasmid 888-76-1 (GenBank accession number CP019446.1) and the Esp_Z chromosome, respectively (Fig. 1). It is an IncF plasmid containing *repFIB* and *psiAB* (12), the VapBC and HicAB toxin-antitoxin systems (13–15), and the VgrG and PAAR proteins of a type VI secretion system (T6SS) (16).

CARD/RGI v5.2.0 identified putative antimicrobial resistance (AMR) genes, including multidrug efflux systems AcrAB-TolC and MdtABC-TolC (17, 18). The OmpR/EnvZ osmoregulation pathway was found using KASS (12, 13, 19, 20).

### Data availability.

Complete genome sequences and raw sequence data for Kosakonia cowanii SMBL-WEM22 are available through NCBI under BioProject accession number PRJNA623929 and GenBank accession numbers CP051488 (chromosome) and CP051489 (plasmid).
